# Transcriptome analysis of *Caenorhabditis elegans* lacking heme peroxidase SKPO-1 reveals an altered response to *Enterococcus faecalis*

**DOI:** 10.1093/g3journal/jkaa055

**Published:** 2020-12-30

**Authors:** Yi Liu, Daniel Martinez-Martinez, Clara L Essmann, Melissa R Cruz, Filipe Cabreiro, Danielle A Garsin

**Affiliations:** 1 Department of Microbiology and Molecular Genetics, The University of Texas Health Science Center at Houston, Houston, TX 77030, USA; 2 MD Anderson Cancer Center UTHealth Graduate School of Biomedical Sciences, Houston, TX 77030, USA; 3 MRC London Institute of Medical Sciences, Du Cane Road, London W12 0NN, UK; 4 Department of Medicine, Institute of Clinical Sciences, Imperial College London, Hammersmith Hospital Campus, Du Cane Road, London W12 0NN, UK

**Keywords:** *Caenorhabditis elegans*, heme peroxidase, immunity, *Enterococcus faecalis*

## Abstract

The nematode *Caenorhabditis elegans* is commonly used as a model organism in studies of the host immune response. The worm encodes twelve peroxidase-cyclooxygenase superfamily members, making it an attractive model in which to study the functions of heme peroxidases. In previous work, loss of one of these peroxidases, SKPO-1 (ShkT-containing peroxidase), rendered *C. elegans* more sensitive to the human, Gram-positive pathogen *Enterococcus faecalis.* SKPO-1 was localized to the hypodermis of the animals where it also affected cuticle development as indicated by a morphological phenotype called “dumpy.” In this work, a better understanding of how loss of *skpo-1* impacts both sensitivity to pathogen as well as cuticle development was sought by subjecting a deletion mutant of *skpo-1* to transcriptome analysis using RNA sequencing following exposure to control (*Escherichia coli*) and pathogenic (*E. faecalis*) feeding conditions. Loss of *skpo-1* caused a general upregulation of genes encoding collagens and other proteins related to cuticle development. On *E. faecalis*, these animals also failed to upregulate guanylyl cyclases that are often involved in environmental sensing. Hoechst straining revealed increased permeability of the cuticle and atomic force microscopy exposed the misalignment of the cuticular annuli and furrows. These findings provide a basis for better understanding of the morphological as well as the pathogen sensitivity phenotypes associated with loss of SKPO-1 function.

## Introduction

The peroxidase-cyclooxygenase superfamily consists of heme-containing peroxidases that are found in all kingdoms of life. Functions for these proteins include roles in the immune response and in tissue development and often involve functional pairing with an NADPH oxidase (Zamocky *et al.* 2008). NADPH oxidases produce reactive oxygen species (ROS) including H_2_O_2_ by either direct or indirect means. Heme peroxidases use H_2_O_2_ in further reactions that can include the generation of more potent oxidants such as HOCl and OSCN^−^ upon reaction with Cl^−^ and SCN^–^, respectively, or the formation of tissue-hardening, di-tyrosine crosslinks between extracellular matrix proteins, or by acting as a catalase to simply reduce H_2_O_2_ to water [Reviewed by Lambeth (2004) and Rada and Leto (2008 )]. Perhaps the best-studied heme peroxidase is myeloperoxidase found in the phagosomes of mammalian innate immune cells such neutrophils, which generates HOCL following the oxidative burst created by the NADPH oxidase, Nox2. However, these proteins can also play a role in barrier defense as exemplified by lactoperoxidase, which generates oxidants in both the respiratory and gastrointestinal mucosae by using the H_2_O_2_ produced by dual oxidases (Lambeth 2004; Rada and Leto 2008).

*Caenorhabditis elegans* is an established model for studying host-pathogen interactions and produces ROS via an NADPH oxidase called BLI-3 following microbial infection (Chávez *et al.* 2009). However, BLI-3 also plays a role in worm development as the source of ROS necessary for cross-linking the collagen proteins to form the outer cuticle (Edens *et al.* 2001). Consistent with its role in cuticle development, BLI-3 is found in the hypodermis of the worm, but is also present in the intestine and in the pharynx (Edens *et al.* 2001; van der Hoeven *et al.* 2015). The NADPH oxidase domain of this dual oxidase generates the H_2_O_2_, whereas the heme peroxidase domain uses the H_2_O_2_ to generate the tyrosine radicals (Edens *et al.* 2001). Interestingly, the BLI-3 peroxidase domain is not the only peroxidase involved in this process. Separate peroxidases, such as MLT-7, also participate in the proper formation of the worm cuticle (Thein *et al.* 2009).

MLT-7 is one of 12 potential heme peroxidases encoded in the *C. elegans* genome (Thein *et al.* 2009; Tiller and Garsin 2014). We previously showed that loss of two other peroxidases, SKPO-1 and HPX-2 increased susceptibility to infection by *E. faecalis* (Tiller and Garsin 2014; Liu *et al.* 2019). Loss of *skpo-1*, but not *hpx-2*, resulted in a morphology phenotype called “dumpy” in addition to the susceptibility phenotype (Tiller and Garsin 2014). Dumpy phenotypes are associated with changes in cuticle structure. Although loss of *hpx-2* did not result in any obvious morphological changes, staining with Hoechst dye indicated that the cuticle was more porous compared to wild-type animals (Liu *et al.* 2019). Therefore, loss of these peroxidases appears to affect cuticle development in addition to increasing pathogen susceptibility (Tiller and Garsin 2014; Liu *et al.* 2019).

Herein, we present transcriptome analysis of the *skpo-*1 loss-of-function mutant in order to understand how gene expression is altered in this genetic background and how these changes might impact its phenotypes in response to both non-pathogenic *E. coli* and pathogenic *E. faecalis* bacterial strains. Our analysis showed that exposure to *E. faecalis* induced a strong transcriptional response in both wild-type N2 and *skpo-1* mutant worms. Yet, a defined transcriptional response dependent on the *skpo-1* genotype was additionally observed. In particular, we saw significant changes in gene expression related to cuticle formation and a failure to upregulate genes encoding neurosensory guanylyl cyclases on *E. faecalis*. Additional functional characterization of the *skpo-1* cuticle defect was performed using Hoechst staining and atomic force microscopy (AFM), a technique recently adapted to *C. elegans* to acquire topographical information under physiological conditions at nanometer resolution (Essmann *et al.* 2017, 2020). Overall, our analysis identified genes and structural features that might underlie *skpo-1* animal’s morphological defects as well as its sensitivity to a human pathogen.

## Materials and methods

### Strains and growth conditions

For general maintenance, *C. elegans* strains were grown at 20°C *E. coli* OP50 (Brenner 1974) that was spotted onto Nematode Growth (NG) agar plates as previously described (Hope 1999). The wild-type parent strain was N2 (Brenner 1974). The *skpo-1* mutant strain was GF89, containing the deletion allele [*skpo-1* (ok1640) II] (Tiller and Garsin 2014). Exposure of *C. elegans* to *E. faecalis* strain OG1RF (Dunny *et al.* 1978), was carried out as originally described (Garsin *et al.* 2001). To generate the plates *E. faecalis* was grown in Brain Heart Infusion (BHI) medium for 5 h and seeded (10 µl) onto BHI agar plates (gentamycin 50 µg/ml) and incubated at 37˚C for 24 h. To synchronize the animals for pathogen exposure followed by RNA extraction, L1 stage worms on starved plates were washed off, filtered through a 10 μm filter (pluriSelect, pluriStrainer 10 μm), harvested by centrifugation, transferred to seeded NG plates, and grown to the L4 stage. To carry out the exposure prior to RNA sequencing, the L4 animals were seeded onto fresh *E. coli* and *E. faecalis* plates and incubated at 25°C for 16 h before RNA extraction.

### RNA sequencing and data analysis

Animals were washed off the plates, and total RNA was immediately prepared using Trizol (Invitrogen) according to the manufacturer’s instructions. 3-4 biological replicates of N2 and the *skpo-1* mutant exposed to either *E. coli* or *E. faecalis* were prepared. Illumina Hiseq 4000 sequencer with 75 nt pair-ended read format was used to conduct the sequencing. The sequencing reads were quality evaluated and adaptor trimmed using FastQC and Trimmomatic v.0.36 (Bolger *et al.* 2014), with a Phred cut-off value of 25. Following this quality analysis, only a very small number of reads were dropped, 0.07–0.12%, resulting in coverage ranging from 12 to 28 million reads/sample. The resulting reads were mapped to the reference transcriptome, version WBcel235 downloaded from http://ensemblgenomes.org, using salmon v.1.2.1 (Patro *et al.* 2017), with options validateMappings and gcBias. Downstream differential expression analysis was performed in R version 3.6.3 using DESeq2 v.1.26.0 (R Core Team 2018), tximport v.1.14.2 and tidyverse v.1.3.0 libraries (Love *et al.* 2014; Soneson *et al.* 2015; R Core Team 2018; Wickham *et al.* 2019), and Venn diagrams were produced using the VennDiagram library v.1.6.20. Log 2-fold change (log2FC) differences between comparisons were shrunken for visualization and analysis with the ‘ashr’ library v. 2.2-47 (Stephens 2016). To control for multiple comparisons, *P*-values were corrected using Benjamini-Hochberg multiple testing correction within the DESeq2 library to generate adjusted *P*-values, also known as *Q*-values. The differential analysis under the different conditions is tabulated in Supplementary Table S1. To compare samples via principal component analysis (PCA), the counts were transformed using the regularized logarithm function within the DESeq2 library to avoid biases due to high variance in the data.

For gene set enrichment analysis, we filtered the genes that had an absolute value of log2FC less than one to extract the features that had a major impact on host phenotype. Transcripts up or downregulated were analyzed separately. Gene set categories were extracted using WormCat, a high quality and manually curated database for *C. elegans* based on GO annotations, using their webserver (Holdorf *et al.* 2020). KEGG pathway enrichment was done with the goseq library v. 1.38.0 (Young *et al.* 2010). Functional annotations resulting from both enrichments were merged, analyzed, and represented in R, filtering categories according to the adjusted *P*-value (*Q*-value) for multiple comparisons. The WormCat enrichment analysis is tabulated in Supplementary Table S2, and KEGG pathways enrichment is tabulated in Supplementary Table S3.

### Hoechst staining

Hoechst staining of *C. elegans* was performed as previously described with the following modifications (Moribe *et al.* 2004). Specifically, animals were exposed to *E. coli* or *E. faecalis* as described in the “Strains and growth conditions” section. The worms were washed off plates with M9 worm buffer (M9W) (Hope 1999) for three washes, and then incubated in M9W containing 50 µg/ml Hoechst 33258 dye (Invitrogen, cat. no. H1398) at room temperature for 30 min followed by three more washes with M9W. The worms were anesthetized with 25 mM Tetramisole hydrochloride (Sigma, cat. no. L9756-5G) for 10 min before imaging. Images were acquired using an Olympus FLUOVIEW FV3000 confocal microscopy equipped with Fluoview FV315-SW software. Z-stack images were acquired using a step size of 0.7 µm and processed using Olympus cellSens Dimension Desktop software. Hoechst-positive worms were scored based on staining of the cell nuclei, indicative of cuticle penetration. Groups of at least 15 animals were tested on each condition and five biological replicates of the experiment were performed.

### Atomic force microscopy

Samples were prepared and imaged using a JPK NanoWizard3 AFM as previously described (Essmann et al 2017). Briefly, worms for measurements were staged to be day 1 young adults by picking 30-40 L4 stage wild-type and *skpo-1* mutants the day before the measurement. 1-day old worms were paralyzed for 1 h in 15 mg/ml 2,3-butanedione monoxime (BDM), fixed to a 4% agarose bed using tissue glue (Dermabond) and imaged under aqueous conditions in M9 buffer in contact imaging mode (set point 0.3 V, scanning speed 0.8-0.6 Hz, 256 pixel) using type qp-CONT-10 (nanosensors) cantilevers. Images were analyzed using the JPK analysis software by applying line flattening, and by extracting data for line histograms which are displayed using the GraphPad program Prism6.

### Brood size

To measure the brood size of the worms, 9 L4 stage worms were singled on NGM plates, allowed to lay eggs and transferred to a new plate each day until no more eggs were produced. The offspring on the plates were counted to calculate brood size.

### Data availability

The raw sequencing data are available from the GEO database under accession number GSE153088. Supplementary Figures S1–S5 and Supplementary Tables S1–S3 are

available at figshare DOI: https://doi.org/10.25387/g3.13143596.

## Results and discussion

### The *skpo-1* genotype alters gene expression on both *E. coli* and *E. faecalis*

To understand how exposure to *E. faecalis* and the *skpo-1* genotype contributes to changes in gene expression, we exposed N2 and *skpo-1* mutant animals at the L4 stage to *E. coli* (OP50) or *E. faecalis* (OG1RF) for 16 h prior to RNA extraction for RNA-seq. Gene expression following *E. faecalis* exposure was compared relative to *E. coli* exposure for both N2 and the *skpo-1* mutant animals. Additionally, gene expression based on the *skpo-1* genotype compared to N2 was measured for both *E. faecalis* and *E. coli* exposed worms*.* Gene expression was considered significantly changed when the |log2FC| ≥ 1.0 (twofold) and the adjusted significance (*Q*-value) was *P* < 0.05 (Supplementary Table S1). Principal component analysis (PCA) showed a large separation between the groups on the x-axis based on the bacterial exposure indicating that many genes had differential expression levels when exposed to *E. faecalis* compared to *E. coli* (Supplementary Figure S1). The worm genotype, *skpo-1 vs* N2, was the second most important contributor to gene expression differences, as shown by the groupings along the y-axis. The groupings of the genotypes were relatively tight with only some overlap.

**Figure 1:**
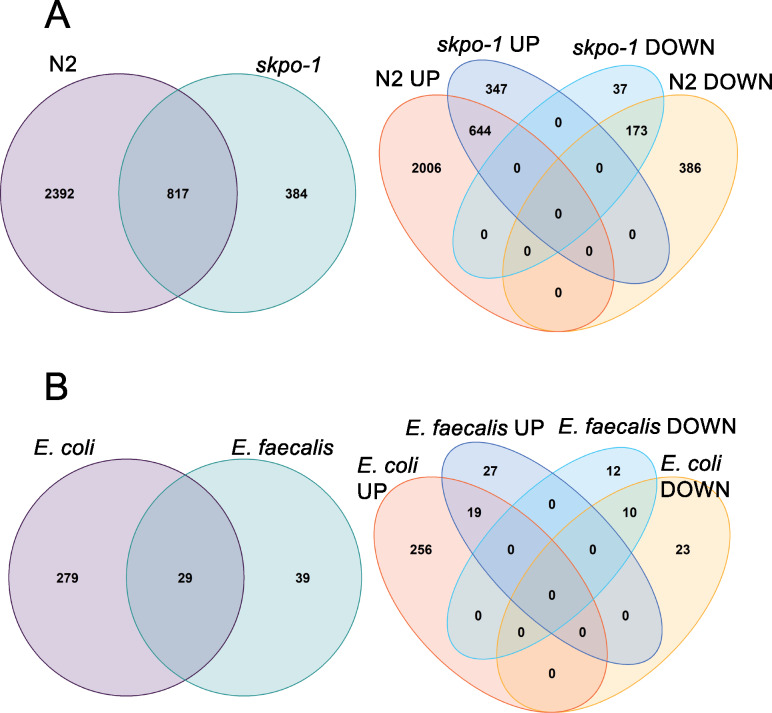
The number of genes with expression changes. (A) The number of genes with significant changes in expression (|log2FC| ≥ 1, *Q* *<* 0.05) in animals exposed to *E. faecalis* relative to those exposed to *E. coli* for N2 and the *skpo-1* mutant. The original data are in Supplementary Table S1. (B) The number of genes with significant changes in expression (|log2FC| ≥ 1, *Q* *<* 0.05) in the *skpo-1* mutant background relative to N2 when exposed *E. coli* and *E. faecalis.* The original data are in Supplementary Table S1.

Figure 1A examines the number of genes that were differentially regulated and the direction of change of *E. faecalis* relative to *E. coli* for both the *skpo-1* mutant and N2 animals. As expected, many genes changed their expression, with more striking differences observed in the N2 background, 3209 in comparison to 1201 (Figure 1A and Supplementary Table S1). In respect to the direction of change, the majority of genes were upregulated in both genetic backgrounds (N2, 2650/3209 = 83%; *skpo-1*, 991/1201 = 83%) (Figure 1A). Overall, the *skpo-1* response to pathogen appeared muted compared to N2. However, the gene expression changes observed in the *skpo-1* mutant were moderately correlated with the wild-type response with an R^2^ = 0.4971 (Supplementary Figure S2A).

Next, we examined the number of genes and in which direction expression changed based on the *skpo-1* genotype relative to N2 (Figure 1B) exposed to the two bacterial diets. 308 and 68 genes were differentially regulated on non-pathogen and pathogen, respectively (Figure 1B and Supplementary Table S1). Somewhat surprisingly, the expression of only 29 genes were affected on both *E. coli* and *E. faecalis* (Figure 1B), with the calculated correlation coefficient of R^2^ = 0.1857 being weak between the two species (Supplementary Figure S2B). Most of the differentially expressed genes were upregulated, 275/308 = 89% on *E. coli* and 46/68 = 68% on *E. faecalis*. Further analysis of these differentially expressed genes and how they might contribute to the *skpo-1* mutant’s phenotypes is detailed next.

### Genes encoding collagen proteins/extracellular material are upregulated in the *skpo-1* mutant or following exposure to *E. faecalis*

**Figure 2:**
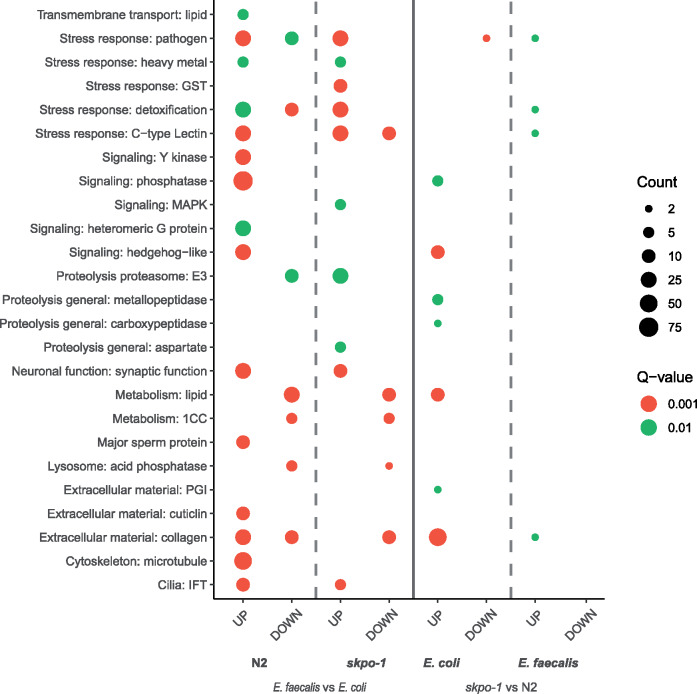
WormCat analysis of genes with significant changes in regulation. Category 2 enrichment annotations are shown for genes that had a |log2FC| ≥ 1 and a *Q* *<* 0.05. The size of the dot represents the number of genes associated with a category. The color represents the strength of the enrichment significance with green indicating a *Q* < 0.01 and red indicating a *Q* < 0.001. The full analysis is in Supplementary Table S2.

To identify functional categories associated with environment and genotype interactions, gene enrichment categories were examined using the WormCat tool (Holdorf *et al.* 2020) for the four different conditions (Figure 2 and Supplementary Table S2). WormCat divides the genes into categories at different levels of refinement, with category 1 encompassing the most general divisions, category 3, the most defined, and category 2, in between (Supplementary Table S2). Focusing on category 2, which was the most insightful (Figure 2), we observed that on *E. coli* genes belonging to the “extracellular material: collagen” category were the most significantly enriched group of genes that changed as a result of the *skpo-1* mutant background. The result is congruent with the knowledge that SKPO-1 is involved in cuticle development, a structure mostly comprised of cross-linked collagen proteins (Tiller and Garsin 2014). Thus the upregulation of the genes encoding the collagen proteins could be an attempt to compensate for cuticle deficits related to the loss of SKPO-1, which is involved in cross-linking the collagen proteins of the cuticle (Tiller and Garsin 2014).

**Figure 3:**
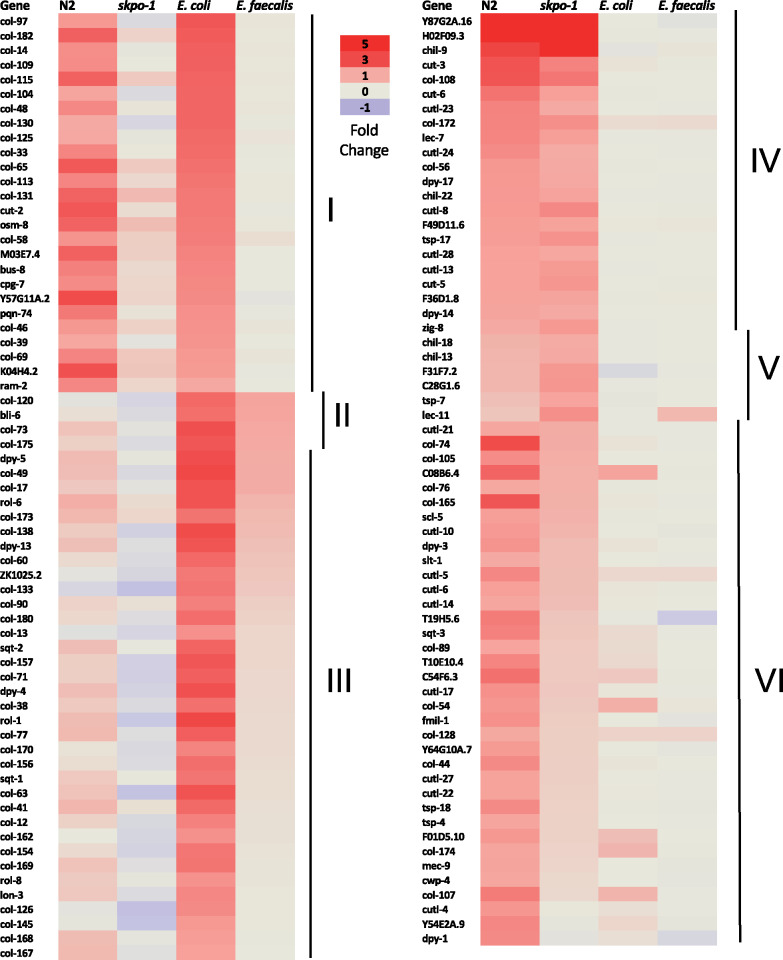
Heat map of genes encoding extracellular matrix proteins that changed significantly in at least one condition. The values of the genes in the extracellular material category that changed significantly (|log2FC| ≥ 1, *Q* *<* 0.05). The first two columns are the changes based on *E. faecalis* exposure relative to *E. coli* for N2 and *skpo-1* mutant animals. The third and fourth column are the changes in the *skpo-1* mutant animals relative to the N2 animals on *E. coli* and *E. faecalis*, respectively*.* Lines labeled with Roman numerals encompass groups of genes with the following features. I) Genes upregulated in N2 animals on *E. faecalis* relative to *E. coli* and in *skpo-1* mutant animals relative to N2 on *E. coli.* II) Genes upregulated in *skpo-1* mutant animals relative to N2 on *E. coli* and *E. faecalis.* III) Genes only upregulated in *skpo-1* mutant animals relative to N2 on *E. coli.* IV) Genes upregulated in N2 and *skpo-1* mutant animals on *E. faecalis* relative to *E. coli.* V) Genes upregulated only in *skpo-1* mutant animals on *E. faecalis* relative to *E. coli.* VI) Genes upregulated only in N2 animals on *E. faecalis* relative to *E. coli.*

Interestingly, this extracellular material WormCat category was much less enriched and significantly changed in the *skpo-1* mutant animals compared to N2 grown on *E. faecalis.* Only 4 genes (group II) were significantly upregulated compared to 65 (group I, II, and III) on *E. coli* (Figure 3). The result is likely explained by the fact that many of these genes were also upregulated on *E. faecalis* (Figure 3, group I). If both *E. faecalis* exposure and the *skpo-1* mutant background upregulate the same gene category, the increases in gene expression occurring as a result of these conditions will be cancelled out when differential expression analysis is applied. The effect can also be observed in that genes in this category are less significantly upregulated in the *skpo-1* mutant background compared to N2 when the animals are exposed to *E. faecalis* (Figure 3, compare the N2 column to the *skpo-1* column). Only 27 genes (group IV and V) are upregulated in the *skpo-1* mutant compared to 84 in the N2 background (group I, IV and VI). Genes encoding extracellular matrix proteins such as collagens were also noted to be enriched upon exposure to *E. faecalis* OG1RF in earlier studies, and overall, many of the same categories were affected when these previous data sets were analyzed using WormCat (Supplementary Figure S3, A and B) (Engelmann *et al.* 2011; Liu *et al.* 2019). There was not as much overlap when a different strain of *E. faecalis* was used, MMH594, which unlike OG1RF produces a cytolysin and might kill by a more toxin-mediated mechanism (Supplementary Figure S3C) (Yuen and Ausubel 2018).

### Genes encoding hedgehog-like proteins are upregulated in *skpo-1* mutant animals or following exposure to *E. faecalis*

Another significantly enriched category belongs to the hedgehog-like signaling factors (Supplementary Figure S4). 12 genes in this category were upregulated in the *skpo-1* mutant background relative to N2 on *E. coli*, but none on *E. faecalis* (Supplementary Figure S4, groups I and III). N2 exposure to *E. faecalis* also altered the expression of these genes, with 25 being upregulated when N2 was exposed (Supplementary Figure S4, groups I, IV and VI). Similar to the expression pattern of the collagen genes, fewer were upregulated when the *skpo-1* mutant was exposed *E. faecalis* relative to *E. coli* (8 genes, Supplementary Figure S4, group IV and V). Hedgehog signaling is a key developmental pathway in Drosophila and mammals, contributing critically to patterning of the embryo, limb buds and organs. However, its role has considerably diverged in *C. elegans*, and it does not play such a central role in development (Bürglin and Kuwabara 2006). Rather, most of the *C. elegans* hedgehog-related genes are expressed in the hypodermis and other epithelial cells and the proteins are frequently secreted into extracellular matrices such as the cuticle. Related to their expression in the hypodermis, knock-down of many of these genes results in molting defects and or defects in the embryogenesis of the hypodermis (Bürglin and Kuwabara 2006; Lažetić and Fay 2017). Possibly, SKPO-1 regulates cuticle homeostasis through regulation of Hedgehog signaling gene expression.

### Exposure to *E. faecalis* increases cuticle permeability

A recent study discovered that *Pseudomonas aeruginosa* damages the *C. elegans* cuticle. By electron microscopy, the cuticle was observed to be more wrinkled and thinner following exposure to this pathogen. Additionally, knock-down of some genes encoding collagens increased susceptibility to *P. aeruginosa* (Sellegounder *et al.* 2019). Interestingly, some strains of *E. faecalis* were recently shown to have collagen-degrading activity dependent on two extracellular proteases, GelE (gelatinase) and SprE (serine protease) (Shogan *et al.* 2015). The strain used in this study, OG1RF, produces these proteases, and mutants lacking these genes are attenuated in the worm model (Sifri *et al.* 2002). Based on these previous studies and our current RNA-seq data, we postulated that exposure to *E. faecalis* may damage these barrier tissues.

**Figure 4:**
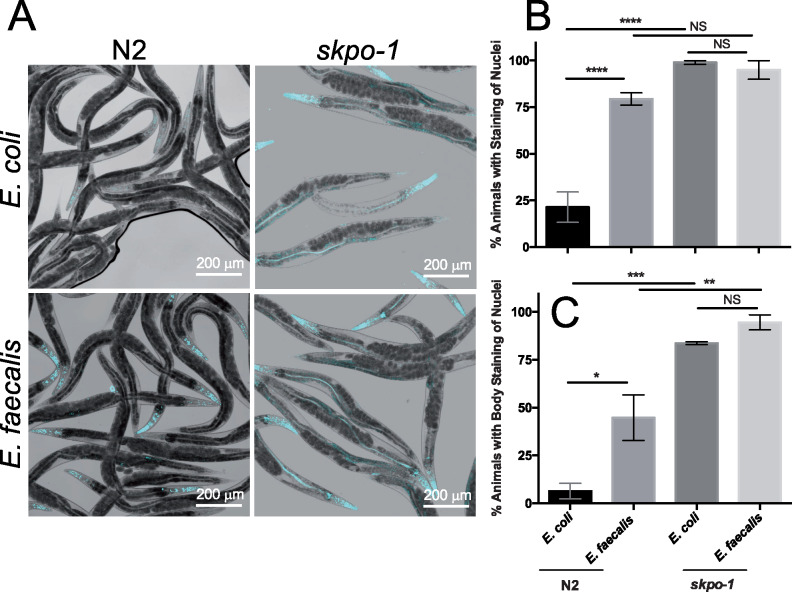
Hoechst staining of nuclei increases as a result of *E. faecalis* exposure and the *skpo-1* genotype. (A) Hoechst staining of nuclei indicative of cuticle permeability in N2 and *skpo-1* mutant animals following exposure to *E. coli* or *E. faecalis.* The representative images are merged pictures taken with bright field and a DAPI/Hoechst emission filter. (B) Quantification of the percentage of animals with evidence of Hoechst nuclear staining anywhere and (C) in the main body of the worm. Error bars represent the SEM, and groups were compared using 2-way Anova. Data are from 5 biological replicates with an *N* ≥ 15, *P < 0.05, **P < 0.01, ***P < 0.001, ****P < 0.0001, NS, not significant.

To address this question, we examined how permeable the nematodes were to the Hoechst dye that stains nuclei. When a population of N2 animals, feeding on *E. coli*, were exposed to the dye, only about 20% had stained nuclei (Figure 4, A and B). As observed previously, most of the staining occurred near the heads of the animals, as a result of nuclear staining in both hypodermal and pharyngeal cells nuclei (Figure 4A) (Liu *et al.* 2019). Recall that the hypodermal cells are the “skin” under the outer cuticle, but that cuticle material also lines the inner surface of the pharynx (Albertson and Thomson 1976). Notably, the overall staining of N2 animals following a 16-h exposure to *E. faecalis* or *E. coli*, was roughly 80% compared to 20%, respectively (Figure 4B). Occasionally, hypodermal nuclei along the body additionally absorbed the stain, as quantified in Figure 4C. If just counting staining along the body there was an increase to 45% on *E. faecalis* from 6% on *E. coli* (Figure 4C). These data suggest that the cuticle became more permeable following *E. faecalis* exposure. We propose that exposure to *E. faecalis* damages the *C. elegans* cuticle and that the observed increase in expression of the hedgehog-like signaling genes and the extracellular matrix genes may represent an attempt to rebuild cuticular material that is under assault.

### Loss of the *skpo-1* gene results in increased cuticle permeability and irregular annuli and furrow structure

Consistent with the observed misregulation of genes involved in cuticle development, we previously observed that *skpo-1* mutant animals have a dumpy phenotype of incomplete penetrance, causing the animals to be small and misshapen (Tiller and Garsin 2014). Dumpy phenotypes are associated with defects in cuticle biogenesis. To see if this results in a cuticle more penetrant to Hoechst dye, we exposed *skpo-1* mutant animals to the dye following feeding on both *E. coli* and *E. faecalis.* Almost all the *skpo-1* mutants had stained nuclei compared to about 20% of the N2 animals fed *E. coli.* Exposure to *E. faecalis* did not further increase the overall staining (Figure 4, A and B). As mentioned above, most of the Hoechst staining occurs near the heads of the animals, with less evidence of cuticle penetration along the main length of the body. However, when just the body was scored (Figure 4C), 80–90% of *skpo-1* mutants exhibited staining independent of bacterial exposure, unlike N2 in which 6% were positive on *E. coli* and 45% on *E. faecalis*.

**Figure 5:**
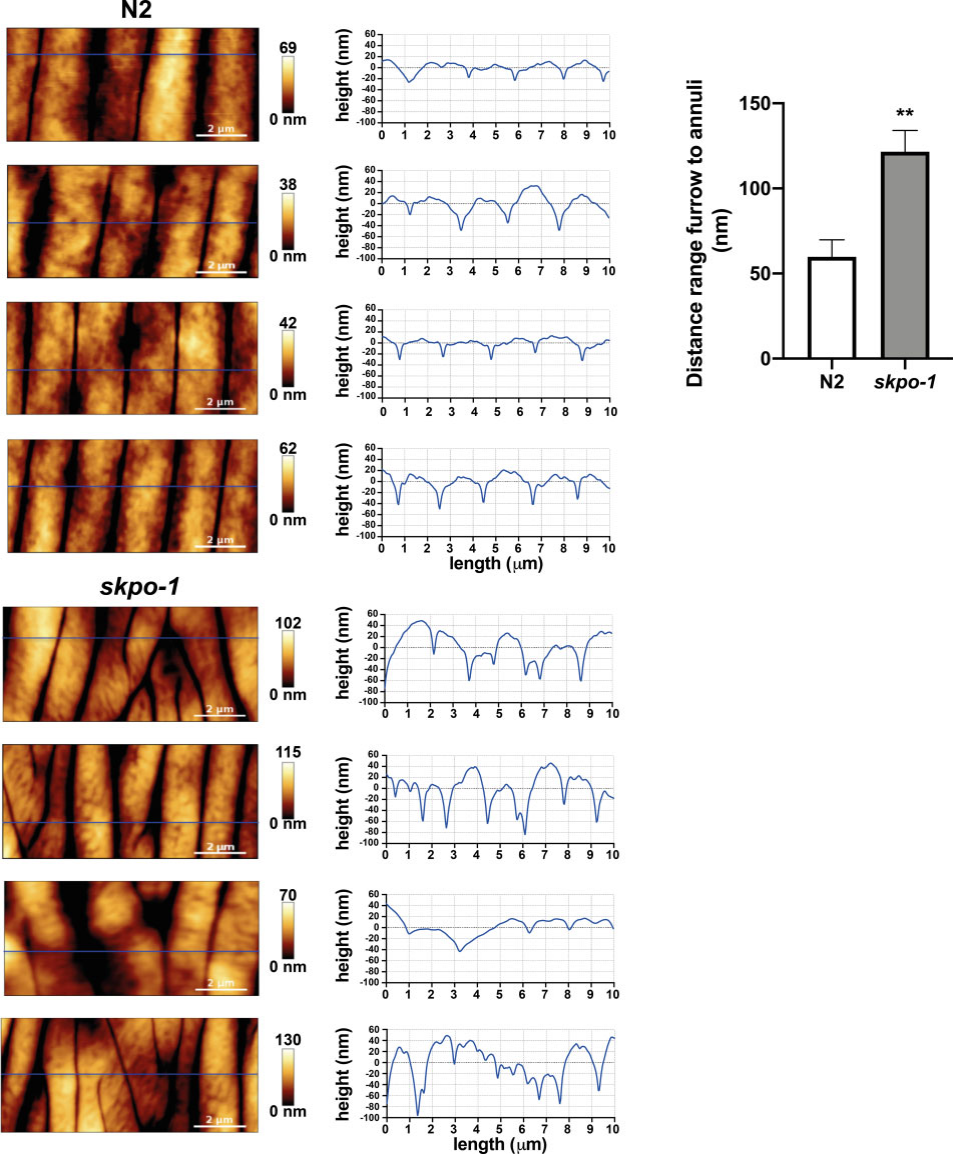
AFM topography images of N2 and *skpo-1* mutant animals. AFM topography images are presented on the left and line histograms on the right of representative AFM analyses of N2 and *skpo-1* mutant animals. These images were taken as cross sections across the cuticle in the neck region of the worms. The dark areas are the furrows that lie in between the lighter, raised regions that are the annuli. Four N2 and *skpo-1* mutant animals were analyzed, and the minimum to maximum distance of the furrow to annuli was taken from each histogram to calculate cuticle depth difference between genotypes in the right panel. A two-tailed, unpaired t-test measured the significance of the difference as *P* = 0.0085. The error bars represent the SEM.

To further study the nature of the *skpo-1* mutant’s cuticular defect, we used atomic force microscopy (AFM), to more closely examine the topography of the major surface structures (Allen *et al.* 2015; Essmann *et al.* 2017). Typical structures of the cuticle include the alae, three parallel ridges along the length of the worm on either side of the body, and a series of regularly separated annuli and furrows running perpendicular to the alae. Imaging the cuticle of wild-type and *skpo-1* mutants by AFM, it was observed that the annuli and furrows were irregularly spaced in the *skpo-1* mutants compared to N2 (Figure 5). To illustrate this, 4 different animals of each genotype were imaged and line histograms, similar to a cross section, were generated from these images. The line histograms generated from the *skpo-1* mutant animals were highly irregular compared to N2 consistent with a malformed cuticle (Figure 5). Although N2 wild-type worms have very regular +/- 2 µm wide annuli interspaced by parallel-oriented furrows, the *skpo-1* mutant animals have furrows that deviate to the left or right rather than running in parallel lines. Consistent with their dumpy phenotype, the average of the maximum range depth between the annuli and the furrows measured for *skpo-1* mutant animals was 2.07-fold greater than N2 animals (Figure 5, right panel). Although our sample size was small, the results indicate that the *skpo-1* mutant’s cuticle is malformed, in agreement with the Hoechst dye experiment and the observed *dpy* phenotype.

Interestingly, *C. elegans* mutants with cuticle defects have previously been reported as having altered stress responses. Specifically, severe disruption of furrows, such as occurs in *dpy-7* and *dpy-10* mutants, were correlated with the activation of the transcription factors SKN-1, ELT-3 and STA-2 to upregulate genes encoding glutathione-S-transferases, *gpdh-1*, and antimicrobial peptides such as *nlp-29*, respectively (Dodd *et al.* 2018). These genes, however, were not upregulated in the *skpo-1* mutant (Supplementary Table S1). Furthermore, the enrichment analysis indicated only subtle changes in the expression of genes in the stress response categories (Figure 2 and Supplementary Table S2). With the caveat that these strains were not compared side-by-side, it appears that the *skpo-1* mutant animals have a different and less severe cuticle irregularity compared to the *dpy-7* and *dpy-10* mutants. In a previous AFM study, the *dpy-7* and *dpy-10* mutants displayed a cuticular defect that manifested itself as a net-like structure on the surface of the animals rather than clearly visible rows of annuli and furrows (Essmann *et al.* 2017). In contrast the *skpo-1* mutant animals still had discernable annuli, but these features failed to lineup in parallel rows due to the irregularity of the furrows (Figure 5).

### Genes associated with spermatogenesis are upregulated in N2, but not in *skpo-1* mutant animals following exposure to *E. faecalis*

We found that certain categories of signaling genes were upregulated only in N2, but not in *skpo-1* mutant animals, when exposed to *E. faecalis.* Categories displaying this pattern of expression, included tyrosine kinases, phosphatases and heteromeric G proteins (Figure 2 and Supplementary Table S2). This expression pattern is distinct from the one belonging to hedgehog-like signaling components, which were found to be upregulated in two conditions—N2 exposed to *E. faecalis* and by deletion of *skpo-1* in non-pathogenic conditions.

**Figure 6:**
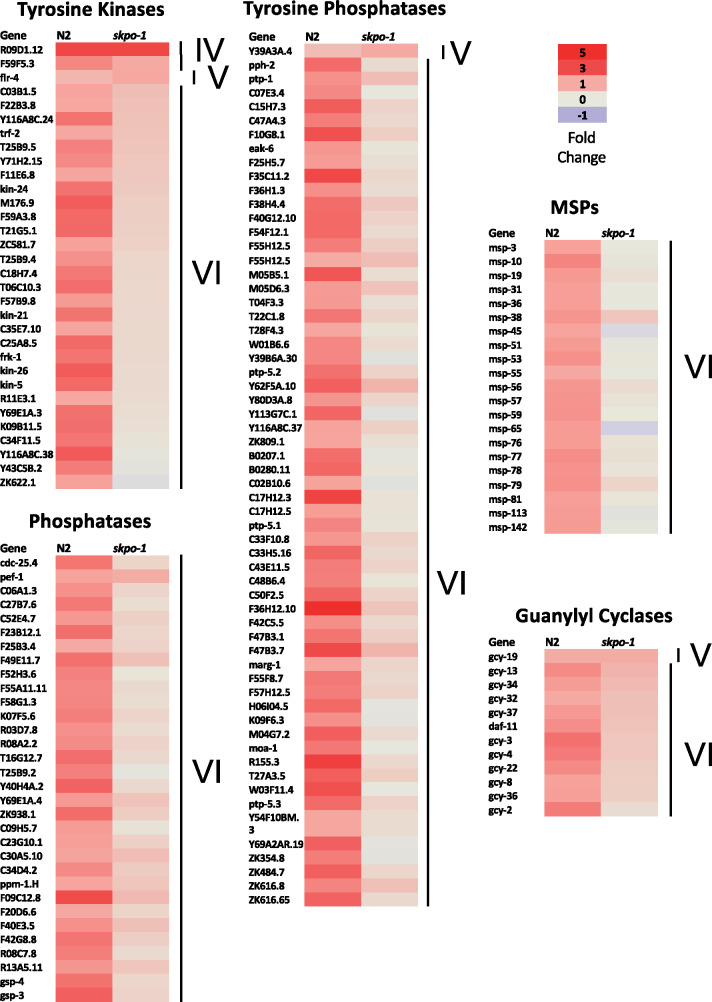
Genes significantly upregulated in N2, but not *skpo-1* mutant, animals when exposed to *E. faecalis.* The values of the genes in the tyrosine kinase, phosphatase, tyrosine phosphatase, major sperm protein and guanylyl cyclase WormCat categories that changed significantly (|log2FC| ≥ 1, *Q* *<* 0.05) following *E. faecalis* exposure relative to *E. coli* for N2 and *skpo-1* mutant animals. Lines labeled with Roman numerals encompass the same groups as originally described in Figure 3. IV) Genes upregulated in N2 and *skpo-1* mutant animals on *E. faecalis* relative to *E. coli.* V) Genes upregulated only in *skpo-1* mutant animals on *E. faecalis* relative to *E. coli.* VI) Genes upregulated only in N2 animals on *E. faecalis* relative to *E. coli.*

To understand the roles of the phosphatase and kinase encoding genes upregulated in N2 animals, but not *skpo-1* animals, on *E. faecalis* (Figure 6 and Supplementary Table S2), we looked at the genes’ expression patterns and phenotypes using WormBase as a resource (Harris *et al.* 2019). A consistent association with expression in the sperm was found for the majority of the genes in these classifications based on a previous microarray analysis in which the global profile of germline gene expression was analyzed in *C. elegans* (Reinke *et al.* 2000). The enrichment of phosphatases and kinases in sperm is thought to arise from reliance on post-translational mechanisms in sperm development (Reinke *et al.* 2000). Additionally, major sperm protein (MSP) was another WormCat category highly upregulated in N2, but not *skpo-1* mutant animals exposed to *E. faecalis* (Figures 2 and 6, and Supplementary Table S2). *C. elegans* sperm resembles amoeboid cells, but instead of using the actin cytoskeleton for motility, the assembly and disassembly of MSP filaments is used (Smith 2006). In addition to these categories of sperm enriched proteins, we observed types such as those associated with sperm-specific (SS) classes and spermatogenesis (SPE) being upregulated in a similar pattern (Supplementary Table S1) (L’Hernault 2006).

Why were proteins upregulated in sperm development enriched in the *E. faecalis* exposed population and only in the N2 background? It is known that *E. faecalis* does not support the development of the younger larval stages, which is why pathogen susceptibility is only assessed starting at the L4 or adult stages (Garsin *et al.* 2001). In this analysis, L4 animals were exposed to *E. faecalis* or *E. coli* for 16 h before RNA extraction. L4 is the stage at which the animals undergo sperm development. Oocytes are produced later during the adult stage (L’Hernault 2006). Although L4 animals are observed to reach adulthood on *E. faecalis* and lay eggs (Garsin *et al.* 2001), we speculate that the process is slowed, and when compared to animals with continual exposure to *E. coli*, they are at an earlier stage of germline development. Similar patterns of sperm-enriched gene expression on *E. faecalis* were observed in previous studies that also used L4 animals (Supplementary Figure S3, A and B) (Engelmann *et al.* 2011; Liu *et al.* 2019), but not in one that began the exposure at the adult stage (Supplementary Figure S3C) (Yuen and Ausubel 2018). As to why the pattern was not observed in the *skpo-1* mutant, we speculate that this is the result of a brood size defect (Supplementary Figure S5), which if arising from less germline cells being produced would obscure this expression pattern. Of note, in our previous study of the *hpx-2* mutant, which has a normal brood size, the pattern of sperm enriched genes being upregulated on *E. faecalis* was also apparent (Liu *et al.* 2019).

### A third of the guanylyl cyclase are upregulated in N2, but not in *skpo-1* mutant animals following exposure to *E. faecalis.*

*C. elegans* guanylyl cyclases are frequently expressed in the neurons and are often involved in sensory processing, including gustation, thermosensation, olfaction, phototransduction, and pathogen sensing (Styer *et al.* 2008; Maruyama 2017). *C. elegans* encodes 34 guanylyl cyclases (Maruyama 2017), 11 of which were upregulated in N2 in response to *E. faecalis*, whereas only one was upregulated in the *skpo-1* mutant background (Figure 6 and Supplementary Table S2). Guanylyl cyclases convert GTP into cGMP, an important secondary messenger. Interestingly, guanylyl cyclases sometimes function in signaling pathways with G-protein coupled receptors (GPCRs) (Marazziti *et al.* 2009), which in *C. elegans* are associated with pathogen sensing and modulation of the immune response (Styer *et al.* 2008; Hoffman and Aballay 2019). In fact, the GPCR, NPR-8, was recently shown to specifically modulate resistance to *P. aeruginosa* through its effects on cuticle gene expression (Sellegounder *et al.* 2019). In an area ripe for future investigation, we postulate that the guanylyl cylases upregulated in N2 animals in response on *E. faecalis* may be involved sensing or responding to pathogen, whereas the *skpo-1* mutant animals are deficient.

## Conclusions and future directions

Overall, this study examined the transcriptome of *C. elegans* with a loss-of-function mutation in *skpo-1*. Consistent with previous knowledge that cuticle formation is abnormal in this mutant, genes encoding extracellular material such as collagen were found to be aberrantly upregulated compared to wild type. Additionally, many of the hedgehog-like genes, with phenotypes and expression patterns linking them to roles in the hypodermis and the extracellular matrix, were also significantly upregulated. Further functional study of the *skpo-1* mutant’s cuticle revealed increased cuticle permeability and significant misalignment of the annuli and furrows. Interestingly, the collagen and hedgehog encoding genes were significantly upregulated in wild-type animals upon exposure to *E. faecalis*. Finally, we observed that the *skpo-1* mutant failed to upregulate guanylyl cyclases involved in sensory processing, suggesting that it might be defective in pathogen sensing or response.

Our previous analysis of the *hpx-2* mutant also revealed a significant upregulation of the cuticle biosynthesis genes on both types of bacteria (Liu *et al.* 2019). However, the transcriptomic changes in the *skpo-1* mutant were much broader and encompassed many more genes, including the hedgehog-like gene family. Another difference between these strains was that genes related to pathogen defense were upregulated in the *hpx-2* background compared to wild type, which was postulated to be due to the higher bacterial load. (Liu *et al.* 2019). In contrast, the *skpo-1* strain accumulates wild-type levels of *E. faecalis* in the gastrointestinal tract (Tiller and Garsin 2014). Although previous analyses found evidence of HPX-2 and SKPO-1 being produced in the hypodermis, HPX-2 is additionally localized to the pharynx, possibly explaining this difference (Tiller and Garsin 2014; Liu *et al.* 2019).

A conundrum that remains unanswered is how the outcome of an infection by a pathogen that colonizes and infects the gut of *C. elegans* affected by a protein primarily associated with the cuticle? Afterall, other proteins involved in cuticle development do not increase pathogen sensitivity. For example, MLT-7, another peroxidase whose loss causes a severe cuticle defect (Thein *et al.* 2009), is not more sensitive to *E. faecalis* (Tiller and Garsin 2014)*.* In contrast, Sellegounder *et al.* (2019) found that knocking down certain collagen genes by RNAi increased sensitivity to pathogen. We offer three possible explanations for how SKPO-1 might be affecting pathogen resistance. 1) Perhaps pathogens characterized as intestinal in the worm are also negatively affecting the animal through effects on the outer cuticle. Recall, that during these infections, *C. elegans* is crawling through a lawn of pathogen. Therefore, the outer surface of the animal is exposed. Although there is no evidence of *E. faecalis* colonizing this surface, the exposure may still be detrimental. The data showing increased cuticle permeability following *E. faecalis* exposure (Figure 4) and previously published data showing changes to the structure and thickness of the cuticle following exposure to *P. aeruginosa* supports this assertion. 2) SKPO-1 is expressed at a low level in the GI tract (pharynx and/or intestine) and its loss weakens the barrier tissue at this location. This assertion is not supported by our previous localization data using a SKPO-1 specific antibody (Tiller and Garsin 2014), but it is possible that a low level of SKPO-1 expression was missed. 3) In previous work on SKPO-1, we observed that loss of SKPO-1 led to high levels of hydrogen peroxide production during infection (Tiller and Garsin 2014). Further KEGG enrichment analysis supports this observation as glutathione metabolism, which plays a key role in redox homeostasis, was found up-regulated in *skpo-1* mutants but not N2 worms when fed *E. faecalis* versus *E. coli* (Supplementary Table S3). Further, we found lysosome and drug metabolism categories to be functionally enriched in both genotypes in response to *E. faecalis*. Interestingly, biosynthesis of unsaturated fatty acids was specifically down-regulated in *skpo-1* mutants but not N2. Recent evidence supports a link between immunity and lipid metabolism with the transcription factor NHR-49/PPAR-α playing a key role in immunometabolism (Dasgupta *et al.* 2020). Perhaps changes in immune signaling and immunometabolism are driven by a *skpo-1* dependent redox signal. In support of this idea, we observed a failure of guanylyl cyclases to be upregulated in the *skpo-1* mutant upon exposure to pathogen (Figure 6).

In conclusion, we propose that shoring up the extracellular matrix in exposed tissue may be an important defense against infection with *E. faecalis* and other pathogens. In terms of exactly how SKPO-1 contributes, these data provide a foundation for future dissection.

## Funding

This work was supported by the National Institute of Allergy and Infectious Diseases of the National Institutes of Health under award numbers R01AI076406 and R01AI150045 to D.A.G. F.C. is supported by the Wellcome Trust/Royal Society (Sir Henry Dale Fellowship - 102531/Z/13/A) and Medical Research Council (MC-A654-5QC80).

*Conflicts of interest*: None declared.
